# Serotonin Receptors in Areas of the Emotion Regulation Network in Human and Rat Brains—A Comparative Autoradiographic Study

**DOI:** 10.1002/cne.70068

**Published:** 2025-07-16

**Authors:** Anika Kuckertz, Ling Zhao, Olga Kedo, Katrin Amunts, Nicola PalomeroGallagher

**Affiliations:** ^1^ Institute of Neuroscience and Medicine 1 (INM‐1) Research Centre Jülich Jülich Germany; ^2^ C. & O. Vogt Institute of Brain Research Heinrich‐Heine‐University Düsseldorf Düsseldorf Germany; ^3^ Department of Psychology, School of Public Policy and Management Nanchang University Nanchang China

**Keywords:** emotion network, human, rat, receptor autoradiography, serotonin receptors, translational research

## Abstract

Serotonergic neurotransmission is crucial for emotion processing and is dysregulated in mood disorders. To analyze the pathophysiology of disease and develop effective pharmacological treatments, the suitability of the rat as a model for translational research must be continuously validated. In vitro receptor autoradiography was used to characterize (dis)similarities of regional and laminar serotonergic 5‐HT_1A_ and 5‐HT_2_ receptor distributions between components of the human emotion regulation network and homologous rat areas, including areas of the lateral prefrontal, orbitofrontal anterior and midcingulate cortices, hippocampal cornu Ammonis (CA) and dentate gyrus (DG), and the accumbens, central amygdaloid, and mediodorsal thalamic nuclei. In both species, mean 5‐HT_1A_ densities were highest in cingulate area 25/infralimbic cortex and the hippocampus, and lowest in the accumbens. Whereas human CA presented significantly higher 5‐HT_1A_ density than DG, the opposite was found in rats. Across the cortical depth, in humans, layers I–III and V contained the highest and lowest 5‐HT_1A_ densities, respectively. In rats, layers I–II contained the lowest and layers V–VI the highest 5‐HT_1A_ values. Mean 5‐HT_2_ densities were lower than 5‐HT_1A_ densities in all areas of both species, whereby layers III and VI contained the highest and lowest 5‐HT_2_ densities, respectively. Rats presented a more widespread range of significant differences concerning the ratio between 5‐HT_1A_ and 5‐HT_2_ receptors across examined areas than did humans. Concluding, this comparative study reveals species differences in 5‐HT_1A_ and 5‐HT_2_ receptor densities in components of the emotion regulation network, which should be considered when using the rat as a model in the translational research of mood disorders.

## Graphical abstract

**Serotonin receptors in areas of the emotion regulation network in human and rat brains a comparative autoradiographic study**. Our systematic comparative analysis revealed significant differences of 5‐HT_1A_ and 5‐HT_2_ receptor distributions and of their balance within and across components of the human emotion regulation network and homolog areas in rats. The data contribute to validate the rat as an appropriate model in the translational research of human mood disorders and provide reference data for comparisons to disorder related alterations.

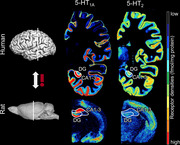

## 1 Introduction

**FIGURE 1:**
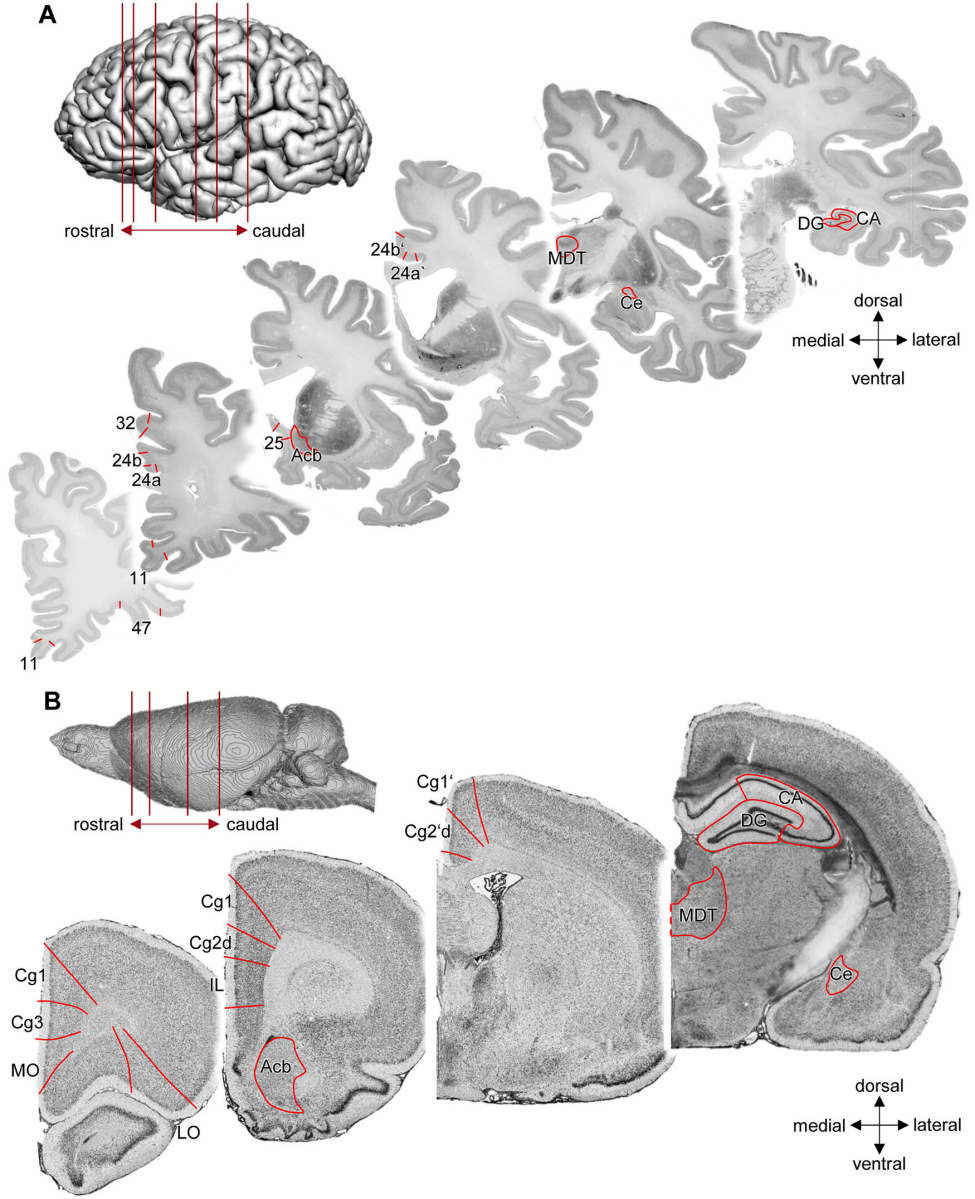
Localization of the investigated human and rat brain areas. (A) Lateral views and serial coronal sections (right hemisphere) of the Big Brain (RRID:SCR_001593) provided by the Human Brain Project (RRID:SCR_002241) (Amunts et al. 2013) and Jülich brain atlas (RRID:SCR_023277) (Amunts et al. 2020) and (B) the Waxholm Space atlas of the Sprague Dawley rat brain (Papp et al. 2014). Red lines indicate the rostrocaudal level from which the exemplary cell body–stained sections were taken to demonstrate the location and cytoarchitecture of the analyzed areas in the human (Amunts et al. 2013) and rat brain (Haghir et al. 2023). Nomenclature of brain regions is provided in Table 1.

Serotonin is involved in the modulation of physiological and behavioral processes such as blood pressure, food intake, and the regulation of emotion and mood (Kohn et al. 2014; Lesch and Waider 2012). The emotion regulation network has been defined on the basis of functional imaging studies and encompasses areas involved in managing and controlling emotional responses. That is, it regulates which emotions arise and when this happens, how long they last, and how these emotions are experienced and expressed (for reviews, see Gross 2014; Morawetz et al. 2020; Palomero‐Gallagher and Amunts 2022; Underwood et al. 2021). The emotion regulation system covers a complex brain network composed of cortical and subcortical structures. Key areas in this network are prefrontal areas 9 and 10, orbitofrontal areas 11 and 47, and cingulate areas 25, 24, 24’, and 32, as well as the hippocampus (Gilbert et al. 2010; Kohn et al. 2014; Palomero‐Gallagher and Amunts 2022; Rolls 2004, 2019; Stevens et al. 2011; Vogt 2005; Wolf et al. 2018) (Figure 1). Subcortical structures most frequently associated with the control of emotions include the accumbens (Acb), central amygdaloid (Ce), and mediodorsal thalamic (MDT) nuclei.

Serotonin, one of the phylogenetically oldest neurotransmitters, exerts its effects via activation of both ionotropic and metabotropic receptors (Pourhamzeh et al. 2022; Żmudzka et al. 2018). There are six families of metabotropic receptors (5‐HT_1_, 5‐HT_2_, 5‐HT_4_, 5‐HT_5_, 5‐HT_6_, 5‐HT_7_), which encompass additional subtypes and, depending on the second messenger systems to which they are coupled, have an excitatory or inhibitory effect. The 5‐HT_3_ receptor is a ligand‐gated Na^+^ and K^+^ ion channel and is thus excitatory in nature. Dysfunctions in serotonergic neurotransmission have been associated with the pathophysiology of mood disorders (Albert and Blier 2023; Asan et al. 2013; Drevets et al. 2007; Hedlund 2009; Pourhamzeh et al. 2022; Rebholz et al. 2018; Tauscher et al. 2001; Walstab et al. 2010; Wang et al. 2016). Neuroimaging studies described altered distribution patterns of 5‐HT_1A_ and 5‐HT_2_ receptors in areas of the emotion regulation network in patients (Arora and Meltzer 1991; Biver et al. 1997; Drevets et al. 1999; Schneck et al. 2021; Wang et al. 2016) and in rat models of depression (Topic et al. 2007; Watanabe et al. 2003; Zaniewska et al. 2010). Hippocampal 5‐HT_1A_ receptor distribution patterns are also altered in patients with anxiety (Drevets et al. 1999; Mayberg et al. 1999). Although there is strong evidence from preclinical rodent models of mood disorders that 5‐HT_3_ and 5‐HT_4_ receptors are involved in depression and anxiety, studies in humans remain inconclusive (Bétry et al. 2011; Gupta et al. 2016; Rebholz et al. 2018; Walstab et al. 2010). Preclinical studies on the role of 5‐HT_5_ receptors in depression have been hampered by the lack of selective ligands, and results concerning the 5‐HT_6_ and 5‐HT_7_ receptors remain inconclusive, since both their activation and blockade have been associated with antidepressant effects (Hedlund 2009; Jastrzębska‐Więsek et al. 2015; Wesołowska and Nikiforuk 2007; Żmudzka et al. 2018).

Thus, pharmacological treatment of mood disorders mainly targets serotonergic receptors with a predominance of the 5‐HT_1A_ and 5‐HT_2_ subtypes, because antidepressant effects, and thus clinical relevance, of other subtypes are not yet fully understood (Artigas et al. 1996; Hedlund 2009; Kaur Gill et al. 2019; Meyer et al. 1999; Pourhamzeh et al. 2022; Roth et al. 2004; Sargent et al. 2000; Walstab et al. 2010).

The success rate of translational neuroscience lies below 1% and thus requires continuous validation of translational models (Austin 2021; Keifer and Summers 2016). Although mice are preferred as genetic models, the rat remains one of the most widely used species for pharmaceutical research (Cryan and Holmes 2005). In this framework, comparative studies are important to establish whether humans and rats present comparable serotonergic innervation and receptor distribution patterns in regions known to be involved in the neuropathology of mood disorders.

Serotonergic innervation patterns have been extensively studied in rodents (Awasthi et al. 2021; Bonn et al. 2013; Cropper et al. 1984; Lidov et al. 1980; Linley et al. 2013; Maddaloni et al. 2017; Moore and Halaris 1975; Oleskevich and Descarries 1990), less in macaques (Berger et al. 1988; O'Rourke and Fudge 2006; Raghanti et al. 2008), and only relatively sparsely in humans (Lew et al. 2019; Raghanti et al. 2008). Although only a few of these studies were comparative in nature, the combined information they provide highlights the existence of regional and laminar differences in the density of serotonergic terminals between rodent, nonhuman primate, and human brains. Higher 5‐HT_1A_ receptor densities were found in the supragranular than in the infragranular layers of humans, and the opposite was described for rats. Furthermore, in humans, hippocampal 5‐HT_1A_ densities (Duncan et al. 1998; Hoyer et al. 1986a; Pazos and Palacios 1985; Pazos et al. 1987a) and serotonergic innervation (Moore and Halaris 1975; Oleskevich and Descarries 1990; Wilson and Molliver 1991) were higher in CA than DG, and the contrary holds true in rats. In contrast, laminar 5‐HT_2_ receptor distribution patterns are comparable in rats (Pazos et al. 1985; Pompeiano et al. 1994) and humans (Burnet et al. 1995; Hoyer et al. 1986b; Palomero‐Gallagher et al. 2013; Pazos et al. 1987b). Furthermore, they did not necessarily cover all areas of the emotion regulation network, and more importantly, they used diverse methods and compounds for the analyses of receptor distribution patterns. Thus, the adequacy of the rat as a model for diseases associated with serotonergic neurotransmission remains a question of interest for translational neuroscience (Valvassori et al. 2013).

To fill this gap, we applied quantitative in vitro receptor autoradiography to characterize the regional and laminar distribution patters of 5‐HT_1A_ and 5‐HT_2_ receptors in key components of the human emotion regulation network (areas 11, 47, 25, 24a, 24b, 24a’, 24b’, and 32, the cornu Ammonis [CA] and dentate gyrus [DG] regions of the hippocampus, as well as the Acb, Ce, and MDT) and compared them with homologous areas of the rat brain (i.e., MO, LO, IL, Cg2d, Cg1, Cg2’d, Cg1', Cg3, CA, DG, Acb, Ce, and MDT, respectively). We aimed to determine whether the pattern of significant differences between homologous areas in the human and rat brain was the same for 5‐HT_1A_ and 5‐HT_2_ receptors. Furthermore, given that each brain area is characterized by a specific receptor balance subserving its function (Zilles et al. 2002), we also aimed to determine whether the ratio between 5‐HT_1A_ and 5‐HT_2_ receptors across these areas was the same in human and rat brains. We provide a quantitative characterization of (dis)similarities in the serotonergic system of humans and rats, enabling assessment of the adequacy of the rat as a model for translational research on disorders pertaining to this neurotransmitter in humans.

## 2 Materials and Methods

### 2.1 Subjects and Tissue Processing

Human receptor data were taken from previously published studies (Kedo et al. 2018; Palomero‐Gallagher et al. 2015, 2020, 2008; Palomero‐Gallagher and Zilles 2009, 2019; Zilles and Palomero‐Gallagher 2017) and included six human brains (three males, three females; 61–79 years of age) obtained from the body donor program of the Department of Anatomy of the University of Düsseldorf, Germany, in compliance with their Ethics Committee. Donors had no history of neurological or psychiatric disorders or long‐term drug treatment.

Three adult male Wistar rats were anesthetized by isoflurane inhalation and decapitated, and then, their brains were extracted. Animal procedures were performed in accordance with the institutional animal welfare committee at the Research Centre Jülich, Germany (RRID:SCR_023416) and the European Union (National Institutes of Health) guidelines for the use and care of laboratory animals.

Brains were shock‐frozen in isopentane (−50°C) and stored at −80°C until further processing. Two of the brains were serially sectioned coronally and the third in the sagittal plane (thickness 20 µm) using a cryostat microtome (Leica Microsystems, Germany, RRID:SCR_008960), and the sections were thaw‐mounted on glass slides. Neighboring sections were used for the visualization of cell bodies (Merker 1983; Palomero‐Gallagher et al. 2008) and receptors.

### 2.2 Receptor Autoradiography

Binding experiments for 5‐HT_1A_ and 5‐HT_2_ receptor visualization were the same as those performed for human tissue and followed previously published protocols (Palomero‐Gallagher and Zilles 2018).

#### 2.2.1 5‐HT_1A_ receptors

Sections were preincubated for 30 min at 22°C in 170 mM Tris‐HCl buffer (pH 7.7) for rehydration and removal of endogenous substances. The main incubation was performed in 170 mM Tris‐HCl buffer (pH 7.7) plus 0.01 % ascorbic acid and 4 mM CaCl_2_ (monohydrate) for 60 min at 22°C. It comprised parallel incubations with adjacent sections for the visualization of total and nonspecific binding sites. Total 5‐HT_1A_ receptor binding was determined by adding [^3^H]8‐hydroxy‐2‐(di‐*n*‐propylamino)tetralin (8‐OH‐DPAT) (BIOTREND Chemikalien GmbH, Germany, RRID:SCR_012423) (humans: 1 nM, *K*_D_: 0.5 nM; rats: 0.3 nM, *K*_D_: 0.3 nM) to the buffer. Nonspecific binding was determined by adding [^3^H]8‐OH‐DPAT (0.3 nM) plus serotonin (1 µM) as a displacer to the main incubation buffer. Finally, sections were rinsed in 170 mM Tris‐HCl buffer (pH 7.7) for 5 min at 4°C and dipped three times into distilled water for 2 s each at 4°C to stop the binding process and eliminate unbound ligand. [^3^H]8‐OH‐DPAT is a highly selective agonist that labels both, the presynaptically located autoreceptors and the postsynaptic binding sites of the 5‐HT_1A_ receptor (De Vry et al. 2004; Mineur et al. 2015; Pazos et al. 1987a; Sotelo et al. 1990). Additionally, [^3^H]8‐OH‐DPAT has an agonistic effect on the 5‐HT_7_ receptor, though with a much lower potency than at the 5‐HT_1A_ receptor (Hedlund et al. 2004; Lovenberg et al. 1993; Ruat et al. 1993).

#### 2.2.2 5‐HT_2_ receptors

The 170 mM Tris‐HCl buffer (pH 7.7) was used for all experimental steps. Sections were preincubated for 30 min at 22°C. The main incubation was performed for 120 min at 22°. Total binding was determined using [^3^H]ketanserin (BIOTREND Chemikalien GmbH, Germany, RRID:SCR_012423) (1.14 nM, *K*_D_: 1.14 nM), and for the nonspecific binding experiment, the displacer mianserin (10 µM) was also included in the main incubation buffer. Rinsing was performed at 4°C for two 10‐min periods, followed by three dips in distilled water at 4°C for 2 s each. [^3^H]Ketanserin is a prototypical 5‐HT_2_ receptor antagonist, mainly to the 5‐HT_2A_ subtype, and it also recognizes the 5‐HT_2C_ subtype (Sharpley et al. 1994) and the 5‐HT_1C_ receptor (Herndon et al. 1992), though with lower affinities. Additionally, ketanserin displays weak adrenergic α_1_ receptor blocking properties, whereby it reduces the central sympathetic tone and thus exerts a vasodilatory effect (Biegon et al. 1986; Israilova et al. 2002; Pazos et al. 1985, 1987b).

Specific binding was calculated by the difference between total and nonspecific binding and amounted to 95% for both receptor types. Radioactively labeled sections were co‐exposed with [^3^H]‐standards (Amersham, Germany) of known radioactivity concentrations against tritium‐sensitive films for 15 weeks. The ensuing autoradiographs represent regional and laminar binding site distribution patterns.

### 2.3 Digitization of Autoradiographs

Autoradiographs were digitized as 8‐bit images with an in‐plane resolution of 5 µm/pixel size using an image analysis system AxioVision (Zeiss, Germany, RRID:SCR_002677) connected with a digital CCD camera (Axiocam MRm, Zeiss, Germany) and an S‐Orthoplanar 60‐mm macro lens (Zeiss, Germany) to enable densitometric measurement of receptor binding sites (Palomero‐Gallagher and Zilles 2018; Zilles et al. 2002). The co‐exposed [^3^H]‐standards were used to compute nonlinear calibration curves, which were used to linearly transform the autoradiographs of the radiolabeled sections into a concentration of receptor binding sites (in fmol/mg protein) using in‐house developed MATLAB (MathWorks, Inc., Natick, MA, RRID:SCR_001622) scripts. Linearized autoradiographs were pseudo‐color‐coded for visualization purposes (Palomero‐Gallagher and Zilles 2018).

Histological sections from human brains were digitized using a Huron scanner (TissueScope, CA, RRID:SCR_024996) and rat sections using a light microscope (Axio Imager Vario.A2, Zeiss, Germany) combined with a stage device and a CCD camera (Axiocam 506 mono, Zeiss, Germany). All resulting 8‐bit images have an in‐plane resolution of 1 µm/pixel.

### 2.4 Regions of Interest

We investigated regions identified in human functional imaging studies as being part of the emotion regulation network (Kohn et al. 2014; Quirk and Beer 2006; Stevens et al. 2011; Timbie and Barbas 2015; Wager et al. 2003): medial and lateral orbitofrontal cortex (areas 11 and 47, respectively); anterior (25, 24a, 24b, and 32) and midcingulate (24a’ and 24b’) areas; hippocampus (DG and CA); and the subcortical Acb, Ce, and MDT. In the rat brain, we analyzed the homologous areas MO (medial orbitofrontal) and LO (lateral orbitofrontal); areas IL, Cg2d (cingulate area 2, dorsal part), Cg1 (cingulate area 1), and Cg3 (cingulate area 3); midcingulate areas Cg2’d and Cg1’; DG and CA regions; and Acb, Ce, and MDT (Gabbott et al. 1997; Groenewegen 1988; Heilbronner et al. 2016; Lucas‐Neto et al. 2013; Olucha‐Bordonau et al. 2015; Öngür and Price 2000; Price 2007; Vogt et al. 2013; Vogt and Paxinos 2014). We restricted our analysis to the posterior portion of the body of the human hippocampus and its correlate in the rat brain (i.e., the dorsal hippocampus), since depression‐related alterations in hippocampal volume are most pronounced in the posterior than in the anterior hippocampus (Schermuly et al. 2011), and fMRI studies have shown that the functional connectivity between the posterior hippocampus and brain regions involved in emotion regulation is altered in depression (Ge et al. 2019; Tura and Goya‐Maldonado 2023). To ensure a comparable level of granularity to in vivo neuroimaging studies (Meltzer et al. 2004; Wacker et al. 2009; Wang et al. 2016), we here only report the mean density over the entire CA region, and we do not distinguish between the core and shell portions of the Acb.

Regions of interest were identified according to previously established cytoarchitectonic criteria (Haghir et al. 2023; Palomero‐Gallagher and Zilles 2015; Paxinos and Watson 2013; Vogt and Paxinos 2014) in the sections processed for visualization of cell bodies (Figure 1) (Amunts et al. 2013, 2020; Haghir et al. 2023). The delineations were then transferred to the adjacent receptor autoradiographs, and mean densities were extracted from the autoradiographs using the in‐house developed software AnaRec (Impieri et al. 2019). Depending on the size of the area, we analyzed three to five sections per brain and receptor type.

Mean receptor densities of all areas were visualized for both species as polar plots, and to facilitate comparisons of the human and rat fingerprints, we used the same axis scaling for each receptor type and identical positions for homologous areas. Relative 5‐HT_1A_ and 5‐HT_2_ receptor densities were calculated for each area as a ratio (%) between the density of the area (*R*_area_) and a receptor type‐related averaged density (*R*_mean_) calculated across all investigated human or rat areas using Equation (1):

(1) $$\mathrm{Ratio}\;\left(\%\right)=\frac{\left(R_{\mathrm{area}}-R_{\mathrm{mean}}\right)}{R_{\mathrm{mean}}}\times 100.$$

### 2.5 Statistical Analyses

The experimental design consists of three variables: species (humans, rats), receptor type (5‐HT_1A_, 5‐HT_2_), and region (13 areas per species). This mixed design analysis of variance was analyzed with linear mixed‐effects models (Baayen et al. 2008). Analyses were performed with the lmerTest package (Kuznetsova et al. 2017) based on the statistical programming environment R (version: 4.0.4) (https://www.r‐project.org/, RRID:SCR_015656).

The linear mixed model was constructed by defining species, receptor type, and region as fixed factors, while subject (brain) was set as a random factor (Equation 2) (Barr et al. 2013; Bates et al. 2015) The resulting omnibus test provided statistical information concerning the main effects of species, receptor type, and region; the interaction effects between them; their fixed effects; and the random effects of subject, which included the random intercept. As the interaction effect between species, receptor type, and region was found to be significant, the simple effects and post hoc test were further analyzed, and the false discovery rate (FDR) approach (Benjamini and Hochberg 1995) was used for multiple comparisons in both cases.

(2) $$RD_{arb}=\alpha_{0}+\alpha_{1}S_{sb}+\alpha_{2}R_{rb}+\alpha_{3}A_{ab}+\alpha_{4}S_{sb}R_{rb}A_{ab}+\alpha_{5}B,$$

where RD is the receptor density, *S* is species, *A* is area, *R* is receptor type, and *B* is brain.

Hierarchical clustering and principal component analyses (PCAs) were performed for human and rat areas separately using in‐house developed MATLAB (MathWorks, Inc., Natick, MA, Rel. R2019a, RRID:SCR_001622) scripts. For equal weighting of the two receptors, densities were normalized by *z*‐scores separately for the 5‐HT_1A_ and 5‐HT_2_ receptors by computing the difference between the density in a given area and the average of that receptor over all areas, then dividing that difference by the standard deviation. Euclidean distances and the Ward linkage algorithm were used to define the degree of (dis)similarity in the hierarchical cluster analyses. The number of stable clusters was determined by *k*‐means analysis combined with the elbow method (Rousseeuw 1987).

## 3 Results

### 3.1 Comparison of Mean Receptor Densities

**FIGURE 2:**
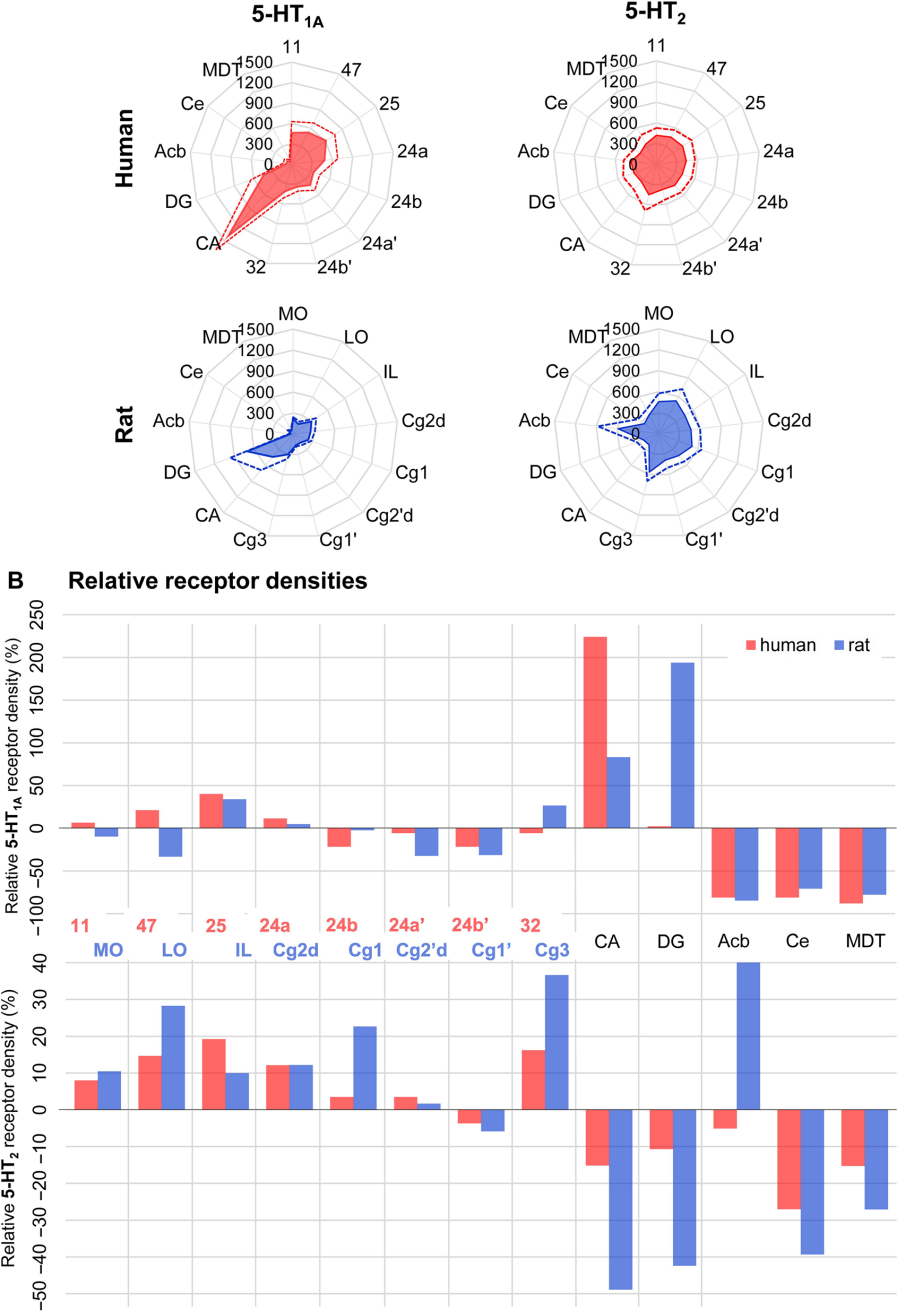
Quantitative values of 5‐HT_1A_ and 5‐HT_2_ receptor distributions. (A) Polar coordinate plots depicting the mean densities (colored surfaces ± standard deviation, dashed lines) in fmol/mg protein of 5‐HT_1A_ (left) and 5‐HT_2_ (right) receptors in the analyzed human (red) and rat (blue) areas. Homologous areas in the human and rat brains are located at the same position in the plot (e.g., human area 11 and its rat homolog MO are both found at “12 o'clock”), and axis scaling is identical for all four plots. (B) Bar charts showing relative 5‑HT_1A_ (top) and 5‑HT_2_ (bottom) receptor densities (in %) across human (red) and rat (blue) areas. Nomenclature of brain regions is provided in Table 1, and numerical values are provided in Tables S1 (for A) and S2 (for B).

The 5‐HT_1A_ and 5‐HT_2_ receptors present regional differences in their expression levels in the human and the rat brain (Figure 2A; Tables S1 and S2). The highest and lowest 5‐HT_1A_ receptor densities were found in the human CA and MDT, respectively, but in the rat DG and Acb, respectively. Concerning the 5‐HT_2_ receptors, the highest and lowest densities were measured in human area 25 and Ce, respectively, and in the rat Acb and CA, respectively. For both species, the magnitude of the difference between the highest and lowest densities is considerably larger for the 5‐HT_1A_ than the 5‐HT_2_ receptors. Consequently, human and rat 5‐HT_2_ receptor fingerprints are relatively “rounder” than those of the 5‐HT_1A_ receptors. This is particularly true for the human brain because in rats, there are two peaks resulting from the relatively higher 5‐HT_2_ density in Cg3 and Acb than in Cg1’, DG, CA or Ce (Figure 2A). In the human brain, 5‐HT_1A_ receptor densities were generally higher than those of 5‐HT_2_ receptors, but the opposite holds true for the rat brain. Additionally, the difference in size between the 5‐HT_1A_ and 5‐HT_2_ receptor fingerprints was larger in the human than in the rat brain (Figure 2A).

In line with these qualitative observations, significant differences were found for both 5‐HT_1A_ and 5‐HT_2_ receptor densities between the human and rat brain (Tables 1, S3, and S4). Specifically, six of the 13 analyzed areas contained significantly higher 5‐HT_1A_ densities in humans compared to rats (Table 1, SET of species; Table S1). Notably, 5‐HT_1A_ density was significantly lower in the human than in the rat DG. Concerning 5‐HT_2_ receptors, we only found a significant difference for the Acb, which presented lower values in humans than in rats.

**TABLE 1:** Results of the statistical analyses determining species‐ or receptor‐specific differences.

Region		SET of species		SET of receptors	
		5‐HT_1A_	5‐HT_2_	Human	Rat
Human	Rat	Human vs. rat	Human vs. rat	5‐HT_1A_ vs. 5‐HT_2_	5‐HT_1A_ vs. 5‐HT_2_
11	MO	0.01	0.77	0.59	0.00
47	LO	0.00	0.39	0.21	0.00
25	IL	0.00	0.79	0.02	0.09
32	Cg3	0.20	0.23	0.31	0.00
24a	Cg2d	0.01	0.82	0.67	0.00
24b	Cg1	0.25	0.27	0.19	0.00
24a‘	Cg2’d	0.00	0.95	0.92	0.00
24b‘	Cg1‘	0.04	0.98	0.39	0.00
CA	CA	0.00	0.15	0.00	0.01
DG	DG	0.01	0.09	0.32	0.00
Ce	Ce	0.73	0.53	0.00	0.01
Acb	Acb	0.51	0.00	0.00	0.00
MDT	MDT	0.96	0.57	0.00	0.00

*Note:* The table shows the results of the simple‐effect tests (Hawrylycz et al. 2011) performed (following the significant results of the Omnibus test, Table S4) with the mixed‐effects model to detect significant (*p* < 0.05) species‐specific (SET of species) or receptor‐specific (SET of receptors) density differences. For the species‐specific comparison, cells highlighted in orange indicate significantly (*p* < 0.05) higher densities in the human than in the rat brain, and cells highlighted in blue signify the opposite situation. For the receptor‐specific comparison, cells highlighted in orange indicate significantly higher (*p* < 0.05) 5‐HT_1A_ than 5‐HT_2_ densities, and cells highlighted in blue signify the opposite situation. The *p*‐values are FDR‐corrected.

**FIGURE 3:**
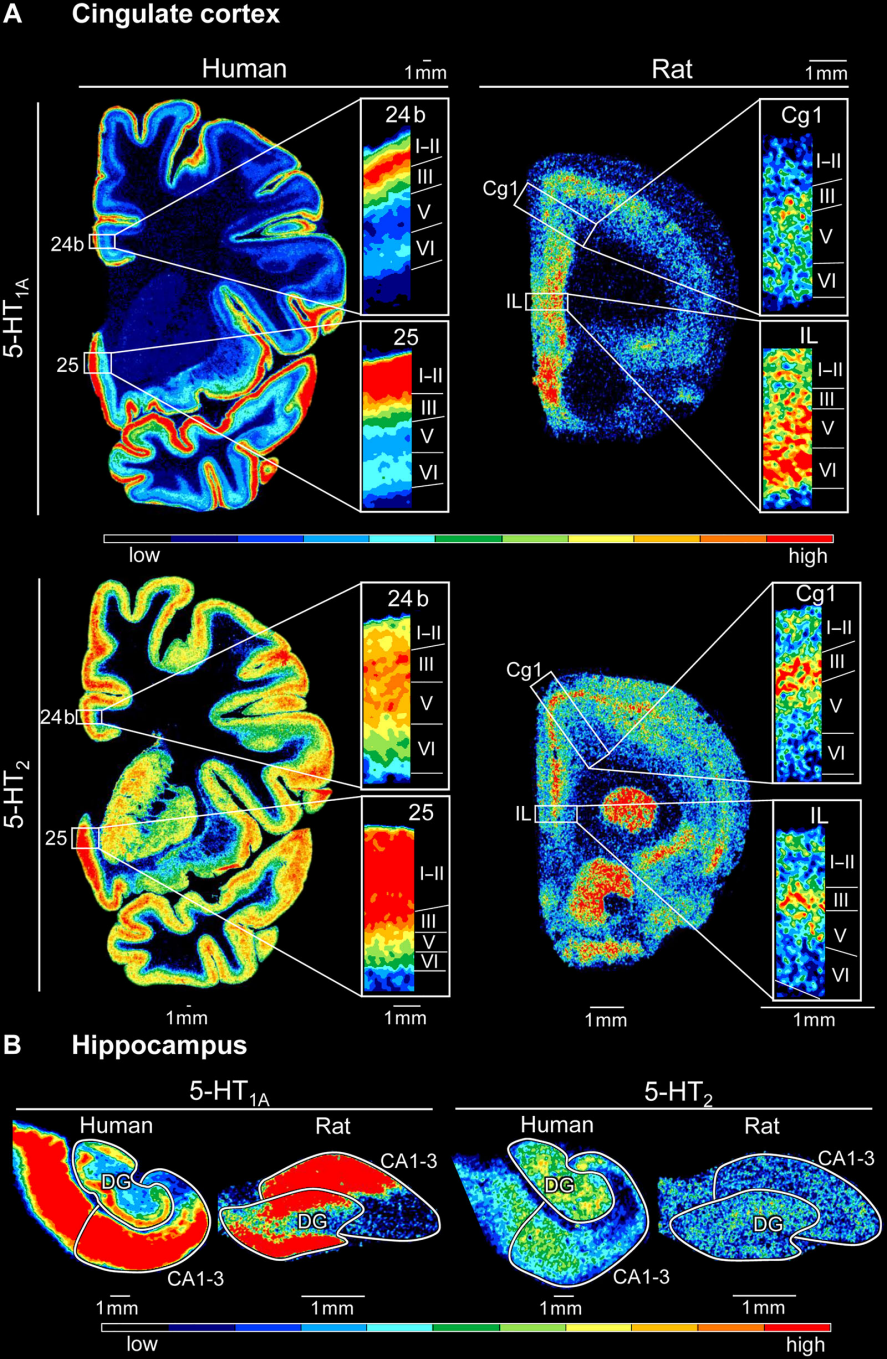
Autoradiograms depicted regional and laminar 5‐HT_1A_ and 5‐HT_2_ receptor distributions. (A) Representative coronal sections showing laminar 5‐HT_1A_ (top) and 5‐HT_2_ (bottom) receptor distribution patterns in human (left) and rat (right) cingulate areas. Laminar distribution patterns of all investigated areas are provided in Figure S1. (B) Representative snippets showing differences in the regional distribution of 5‐HT_1A_ (left) and 5‐HT_2_ (right) receptors within the human and rat hippocampus. Roman numerals indicate cortical layers, and numerical values of receptor densities in fmol/mg protein are provided in Table S1. Nomenclature of brain regions is provided in Table 1.

Concerning the ratio between 5‐HT_1A_ and 5‐HT_2_ receptors across all examined areas, we found a more widespread range of significant differences in the rat than in the human brain (Table 1, SET of receptors). In humans, significant differences were restricted to CA, with a higher density of 5‐HT_1A_ than of 5‐HT_2_ receptors, and the subcortical nuclei, all of which presented lower 5‐HT_1A_ than 5‐HT_2_ receptor densities. In contrast, the rat brain presented significant differences in all examined areas except for IL. Rat CA and DG both contained a higher density of 5‐HT_1A_ than of 5‐HT_2_ receptors, and the opposite held true for the remaining areas. Finally, we determined whether human and rat brains presented comparable relative distribution patterns of 5‐HT_1A_ and 5‐HT_2_ receptors and found a striking species‐specific difference concerning the relative 5‐HT_1A_ receptor densities in CA and DG (Figure 2B). Specifically, significantly higher densities were found in the human CA than the DG, and the opposite held true for the rat hippocampus (Table S4). This was mainly due to the higher 5‐HT_1A_ receptor density in the molecular layer of DG but lower concentration in the CA3 region of rats compared to humans (Figure 3). In contrast, human and rat areas presented a comparable relative distribution pattern of the 5‐HT_2_ receptors, with the notable exception of the Acb, which showed a lower value than the mean in humans but a higher density than the mean in rats (Figure 2A).

### 3.2 Comparison of Laminar Receptor Distributions

Human orbitofrontal and cingulate areas were characterized by the highest 5‐HT_1A_ receptor densities in layers I–III, the lowest values in layers IV (when present) and V, and slightly higher densities in layer VI (Figures 3A and S1). Homologous areas in the rat brain not only showed a principally different 5‐HT_1A_ receptor distribution but also region‐specific variations in this pattern. Areas MO and LO presented a single maximum in layers V and III, respectively, and lower densities in the superficial than the deeper layers. Areas Cg1, Cg2d, and Cg2’d presented a similar 5‐HT_1A_ pattern to that of the orbitofrontal areas, but with a broader maximum, which was centered over layers III and Va in Cg1 and Cg2’d, but encompassed layers III–VI in Cg2d. Area Cg1’ presented a 5‐HT_1A_ receptor maximum in layer V, and its superficial and deeper layers contain comparable values (Figures 3A and S1).

Laminar distribution of 5‐HT_2_ receptors was more similar between humans and rats, with most areas presenting a single maximum centered over layer III and lowest densities in layer VI. Human area 25 constitutes the notable exception, due to a particularly broad maximum that spans layers I–III (Figures 3A and S1). Both species displayed considerable laminar differences within the hippocampus, since human CA presented a distinct laminar distribution, with the highest 5‐HT_2_ receptor densities in the pyramidal layer, and a homogeneous distribution pattern across the rat hippocampus (Figure 3B).

### 3.3 Multivariate Analyses

**FIGURE 4:**
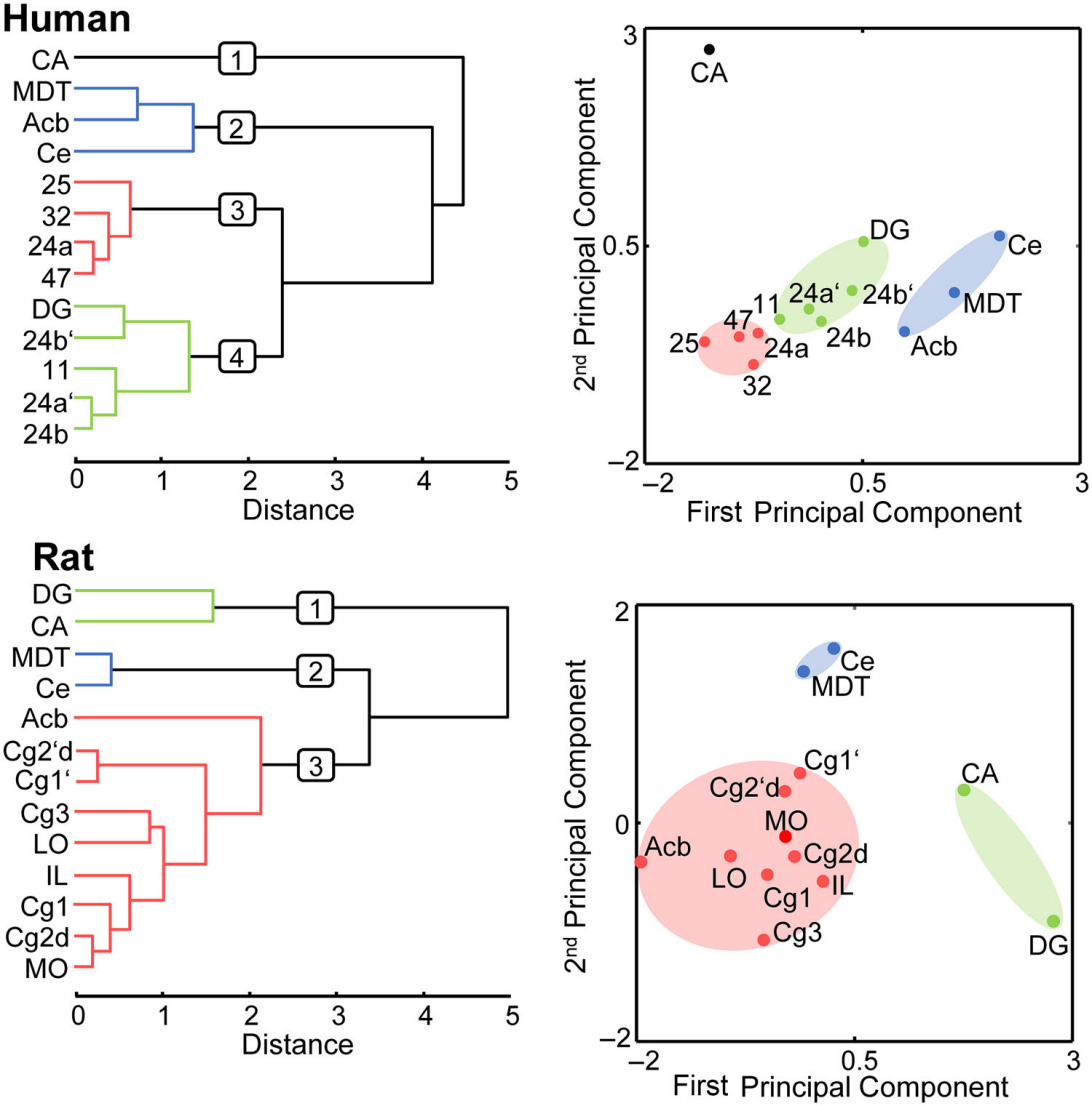
Multivariate analyses of 5‐HT_1A_ and 5‐HT_2_ receptor distributions. Hierarchical clustering (left) and principal component analysis (right) (Ellison‐Wright and Bullmore 2010) to determine clustering of human and rat areas based on (dis)similarities in their serotonin receptor architecture. The *k*‐means clustering revealed four and three as the optimal number of clusters for human and rat areas, respectively. Nomenclature of brain regions is provided in Table 1.

In humans, the hierarchical clustering analysis seems to be driven by the striking differences in the relative 5‐HT_1A_ receptor densities, particularly between the CA and DG. CA was characterized by the highest 5‐HT_1A_ and one of the lowest 5‐HT_2_ receptor densities and segregated from all other areas (cluster 1; Figure 4). This was mainly driven by the second principal component of the PCA, whereas the separation of clusters 2, 3, and 4 was driven by the first principal component. Cluster 2 encompassed the three subcortical nuclei, all of which display very low 5‐HT_1A_ and 5‐HT_2_ receptor densities. Cluster 3 contains lateral orbitofrontal area 47 and the anterior cingulate areas, except for 24b, which was found in cluster 4 together with midcingulate areas 24a’ and 24b’, medial orbitofrontal area 11, and the DG.

In rats, the differences in 5‐HT_1A_ receptor densities between CA and DG were significantly smaller than in humans. Both regions displayed the highest and the lowest 5‐HT_1A_ and 5‐HT_2_ receptor densities, respectively, measured in all examined areas of the rat brain. This similarity in receptor architecture was reflected in their grouping together in cluster 1 separately from the remaining areas, and its segregation was mainly driven by the first principal component of the PCA. The subcortical nuclei MDT and Ce were in cluster 2, whereas the Acb, characterized by higher 5‐HT_2_ densities, was in cluster 3 together with the remaining rat areas. Segregation of clusters 2 and 3 was driven by the second principal component of the PCA and can be explained by the low 5‐HT_1A_ and 5‐HT_2_ densities measured in Ce and MDT.

## 4 Discussion

Our quantitative comparative study systematically analyzed (dis)similarities of 5‐HT_1A_ and 5‐HT_2_ receptor distributions in components of the human emotion regulation network and homologous areas in rats. We provide reliable reference data in healthy brains and contribute to assessing the adequacy of the rat as an appropriate model for translational research of human mood disorders.

### 4.1 Laminar Serotonin Innervation and Receptor Distribution Patterns

Serotonergic neurons are located exclusively in the raphe nuclei of the mammalian hindbrain, but their highly collateralized axons innervate every region of the central nervous system, allowing them to simultaneously modulate numerous brain areas (Soiza‐Reilly and Gaspar 2020). Development of the brain's serotonergic innervation pattern commences during the early embryonal phase and is completed within the first postnatal month (D'Amato et al. 1987; Lidov and Molliver 1982; Wallace and Lauder 1983), and this neurotransmitter system plays an essential role in normal developmental processes (Gaspar et al. 2003; Lauder 1993). However, the density and complexity of serotonergic innervation patterns do not remain static throughout adulthood but are susceptible to changes in brain serotonin levels, thus highlighting the importance of a balanced serotonin homeostasis to preserve serotonergic circuitry (Pratelli et al. 2017).

The few studies addressing serotonergic innervation in the human brain revealed immunoreactive terminals in all analyzed cortical areas, with higher densities in the superficial than the deep layers, and a conspicuous horizontally running plexus in layer I (Raghanti et al. 2008; Trottier et al. 1996). This pattern reflects the distribution we described for the 5‐HT_1A_ receptors, which show uniquely high densities in layers I–III and low values in layers V–VI, as well as that of the 5‐HT_2_ receptors, which present the highest densities in layer III.

The rat cortex receives a dense regional and layer‐specific heterogeneous serotonergic innervation, with a decreasing rostrocaudal gradient in fiber density and highest values in the medial prefrontal cortex (Audet et al. 1989; Beaudet and Descarries 1978; Lidov et al. 1980; Reader 1981; Vertes 1991; Wilson and Molliver 1991), which we also found to contain relatively high 5‐HT_1A_ and 5‐HT_2_ densities. Although in most areas, layers I and VI present the strongest and weakest serotonergic innervation, respectively, we found layer I to present the lowest 5‐HT_1A_ and 5‐HT_2_ densities. Our laminar distribution patterns for both species and receptors are in accordance with previously published autoradiographic and immunohistochemical reports, as well as with the visualization of 5‐HT_1A_ receptor mRNA (Burnet et al. 1995; Chalmers and Watson 1991; Hoyer et al. 1986a, 1986b; Jansson et al. 2001; López‐Giménez et al. 1998; Palomero‐Gallagher et al. 2019, 2008; Palomero‐Gallagher and Zilles 2015; Palomero‐Gallagher et al. 2013; Pazos et al. 1985; Pazos and Palacios 1985, 1987a, 1987b; Pompeiano et al. 1992, 1994; Willins et al. 1997). Interestingly, laminar differences in serotonergic innervation were also found between human and nonhuman primate brains and are thought to indicate a human‐specific shift in the role of serotonin modulation of cortical input and output functions (Raghanti et al. 2008).

Humans and rats present a higher serotonergic axonal density in CA than in DG (Moore and Halaris 1975; Oleskevich and Descarries 1990; Wilson and Molliver 1991). Interestingly, confirming previous observations, we found significantly higher 5‐HT_1A_ densities in CA than in DG of humans and the opposite situation in rats. Our results pertaining to the 5‐HT_2_ receptor are in line with the low detection of 5‐HT_2(A)_ receptor mRNA in both species (Burnet et al. 1995; Pompeiano et al. 1994).

Rat Ce and MDT are almost as strongly innervated by serotonergic neurons as are cortical areas (Asan et al. 2013; Cropper et al. 1984; Vertes et al. 1999) but display some of the lowest 5‐HT_1A_ and 5‐HT_2_ receptor densities measured in the present study, a finding in accordance with previously published reports (Chalmers and Watson 1991; Pazos et al. 1985; Pazos and Palacios 1985; Pompeiano et al. 1992, 1994). In contrast, the Acb presents, together with Cg3, the highest 5‐HT_2_ density in the rat brain. In line with previous studies (Pazos et al. 1985, 1987b), we found a significantly higher 5‐HT_2_ density in the rat than in the human Acb. Also in line with previous publications, we determined comparable laminar 5‐HT_2_ receptor distributions between homologous cortical areas of the human (Hoyer et al. 1986b; Palomero‐Gallagher et al. 2008; Palomero‐Gallagher and Zilles 2009; Pazos et al. 1987b) and rat brain (Pazos et al. 1985; Pompeiano et al. 1994), but considerable differences for the 5‐HT_1A_ receptor (Duncan et al. 1998).

### 4.2 Differential 5‐HT_1A_ and 5‐HT_2_ Receptor Balance in Human and Rat Brains

Brain areas are each characterized by a unique combination of receptor types, their receptor fingerprints. This specific balance between different receptors modulates synaptic connectivity, signal integration, and contribution of the area to network dynamics subserving normal brain function (Palomero‐Gallagher and Zilles 2018; Zachlod et al. 2023; Zilles et al. 2002). Changes in receptor fingerprints are associated with brain disorders such as epilepsy, hepatic encephalopathy, and progressive supranuclear palsy (Chiu et al. 2017; Graebenitz et al. 2011; Palomero‐Gallagher et al. 2012; Palomero‐Gallagher and Zilles 2013), and alterations in serotonergic neurotransmission have been associated with depression (Bartlett et al. 2022; Biver et al. 1997; Drevets et al. 1999; Hrdina et al. 1993; Mann 1999; Schneck et al. 2021; Wang et al. 2016), schizophrenia (Dean et al. 1999; Hurlemann et al. 2005; Joyce et al. 1993; Laruelle 1993; Simpson et al. 1996), and eating disorders (Bailer et al. 2004; Gorwood et al. 2018), highlighting the importance of understanding the relationship between receptor expression levels for the development of effective therapeutic strategies.

Rats are extensively used as animal models, including the field of mood disorders (Planchez et al. 2019), due to their ease of manipulation and well‐characterized behaviors (Hashway and Wilding 2020; Keifer and Summers 2016; Wang et al. 2017). However, we here found humans and rats to differ significantly in the laminar distribution patterns of 5‐HT_1A_ and 5‐HT_2_ receptors. Furthermore, we also found significant differences in the balance between 5‐HT_1A_ and 5‐HT_2_ densities in the majority of analyzed cortical areas, but not in the subcortical nuclei, where both species consistently presented significantly lower 5‐HT_1A_ than 5‐HT_2_ receptor concentrations. The differential balance resulted from the fact that (i) most human areas presented higher 5‐HT_1A_ densities than their rat homologs, but both species contained comparable 5‐HT_2_ densities, and (ii) most rat cortical areas contained significantly lower 5‐HT_1A_ than 5‐HT_2_ densities, which we did not observe in humans.

A further divergent aspect between humans and rats was found when analyzing the relative distribution of 5‐HT_1A_ and 5‐HT_2_ receptors across all examined areas. Here again, the hippocampus was particularly conspicuous because, despite its evolutional conserved neuroanatomy (Zilles 2005), in humans we found a significantly higher 5‐HT_1A_ density in CA than DG, but the opposite held true for rats.

In contrast, we found a good correspondence in the relative distribution patterns of both receptors in human area 25 and its rat homolog IL. Areas 25 and IL were characterized by higher densities of both receptors than the surrounding cortex, thus replicating previously published observations (Palomero‐Gallagher et al. 2008; Pazos et al. 1985; Pazos and Palacios 1985, 1987a, 1987b; Santana and Artigas 2017; Varnäs et al. 2004) and supporting species‐overarching inhibitory functions via 5‐HT_1A_ receptors in areas 25 and IL. We also found a good correspondence concerning relative 5‐HT_1A_ and 5‐HT_2_ distributions in the Ce and MDT, though not in the Acb of both species. Notably, deep brain stimulation of the Acb had antidepressant and antianhedonic effects in patients with treatment‐resistant depression (Bewernick et al. 2010) and in a rat model of depression (Hamani et al. 2012; Lim et al. 2015).

These species‐specific differences in the relative distribution of 5‐HT_1A_ and 5‐HT_2_ receptors resulted in a different clustering pattern of the analyzed areas, with four and three stable clusters identified in the human and rat brains, respectively. The DG clustered with CA in the rat brain but with anterior midcingulate areas in the human brain, and this seems to be driven by the striking species‐specific difference in the relative distribution pattern of 5‐HT_1A_ receptors in CA and DG. Given that DG and CA constitute the main input and output regions of the hippocampus, respectively (Amaral et al. 2007), this clustering pattern could be indicative of a differential involvement of the serotonergic system in the modulation of hippocampal input and output mechanisms in humans and rats, since activation of 5‐HT_1A_ receptors is crucial for the coordination of DG and CA3 neuronal firing during novelty detection processes (Luo et al. 2011; Meeter et al. 2006; Nolan et al. 2011). Interestingly, the human and rat DG differ in the neuroanatomical structure of their principal cell type, that is, the granule cells. Specifically, adult human granule cells have apical and basal dendrites, but rat granule cells only present apical dendrites (Seress and Pokorny 1981; Seress and Mrzljak 1987).

We found the most prominent species differences in the CA and DG regions of the hippocampus, ventromedial area 24, dorsomedial area 32, orbitofrontal area 47, and the Acb. Activation of these areas is associated with behavioral control, the subprocess of emotion regulation enabling the subject to monitor, evaluate, and modulate their emotional response to an external stimulus (Phillips et al. 2008). Cognitive neuroscientists postulated a model of emotion regulation whereby these processes may occur in an automatic (i.e., subconscious) or a controlled (i.e., conscious) manner (Gross 2014; Gross and Thompson 2007; Phillips et al. 2008). The hippocampus and area 32 mediate the so‐called voluntary (conscious) behavioral control, whereas ventromedial area 24, orbitofrontal area 47, and the Acb are involved in automatic behavioral control (Etkin et al. 2011; Merz et al. 2011; Phillips et al. 2008). Interestingly, the cognitive processes associated with voluntary behavioral control are significantly less developed in rodents than the automatic behavioral control strategies, and, with regard to emotion regulation, most differences between healthy controls and patients with major depression are found in areas subserving the automatic regulation of emotions (Rive et al. 2013).

Most important for future studies assessing potential novel drugs in rat models, our findings can provide an explanation as to why compounds such as Vilazodone, Robalzotan, and Gepirone (Arborelius et al. 1999; Drossman et al. 2008; Kaur Gill et al. 2019; Mucke 2000; Zareifopoulos and Dylja 2017) failed in clinical trials and have been discontinued (Hughes et al. 2005; Zareifopoulos and Dylja 2017). They all specifically target the 5‐HT_1A_ receptor, for which we found considerable differences between humans and rats. Thus, it is not surprising that, although they had been classified as highly promising for the treatment of depression after studies in rat models (Arborelius et al. 1999; Kaur Gill et al. 2019; Page et al. 2002), they showed no significant advantage in remission rates and were associated with severe side effects and high placebo response rates (Drossman et al. 2008; Kirsch 2015; Zareifopoulos and Dylja 2017). Thus, our findings question the value of rats as a translational model to test drugs targeting serotonergic receptors. They also highlight the necessity of assessing the adequacy of rats as animal models of disorders involving receptors of other neurotransmitters such as dopamine.

## 5 Conclusions

Concluding, our systematic comparative analysis demonstrates that humans and rats differ significantly in the balance between 5‐HT_1A_ and 5‐HT_2_ receptors both within and across areas involved in the emotion network. The provided quantitative reference data of healthy brains are useful for comparisons with alterations in patients and rat models, including the development of pharmacological treatments. Furthermore, it highlights the necessity of comparative analyses of humans and species commonly used in neuroscientific research as one approach to facilitate and accelerate translational neuroscience.

## Author Contributions

**Anika Kuckertz**: data acquisition, visualization, and interpretation, writing–original draft, writing–review and editing. **Ling Zhao**: data analysis and visualization. **Olga Kedo**: data acquisition and mapping. **Katrin Amunts**: writing–review and editing. **Nicola PalomeroGallagher**: conception and design, data curation and interpretation, writing–review and editing.

## Conflicts of Interest

The authors declare no conflicts of interest.

## Peer Review

The peer review history for this article is available at https://publons.com/publon/10.1002/cne.70068.

## Supporting information




**Supplementary Table 1:** Mean (in fmol/mg protein) 5‐HT_1A_ and 5‐HT_2_ receptor densities ± standard deviations in areas of the emotion regulation network in the human brain and their homologs in rats.

**Supplementary Table 2:** Numerical values of relative 5‐HT_1A_ and 5‐HT_2_ receptor densities (in %) in areas of the emotion regulation network in the human brain and their homologs in rats.

**Supplementary Table 3:** Results of the ANOVA test performed with a mixed‐effects model.

**Supplementary Table 4:** The results of the simple effect test with mixed‐effects model.

**Supplementary Fig. 1:** Laminar 5‐HT_1A_ and 5‐HT_2_ receptor distributions between the human and rat orbitofrontal and cingulate areas.

## Data Availability

Data supporting this study are provided in the Supporting Information.
