# Supplementary material for: Serotonin Receptors in Areas of the Emotion Regulation Network in Human and Rat Brains—A Comparative Autoradiographic Study
Source: J Comp Neurol. 2025 Jul 16;533(7):e70068. doi: 10.1002/cne.70068 (PMC12267679; doi:10.1002/cne.70068)
Supplement: Supplementary file 1 — Supplementary Table 1: Mean (in fmol/mg protein) 5‐HT1A and 5‐HT2 receptor densities ± standard deviations in areas of the emotion regulation network in the human brain and their homologs in rats. [file CNE-533-e70068-s002.pdf]

Supplementary Table 1. Mean (in fmol/ mg protein) 5-HT_{1A} and 5-HT_2 receptor densities ± standard deviations in areas of the emotion regulation network in the human brain and their homologs in rats.

**Species**		**5-HT_{1A} receptors**		**5-HT_2 receptors**	
Human	Rat	Human	Rat	Human	Rat
11	MO	465 ± 163	215 ± 26	421 ± 107	457 ± 120
47	LO	530 ± 156	159 ± 42	447 ± 116	530 ± 190
25	IL	612 ± 157	319 ± 81	465 ± 155	455 ± 112
32	Cg3	412 ± 97	302 ± 65	453 ± 233	565 ± 131
24a	Cg2d	487 ± 193	250 ± 65	437 ± 127	464 ± 130
24b	Cg1	343 ± 95	233 ± 45	403 ± 147	507 ± 141
24a‘	Cg2’d	412 ± 99	162 ± 42	403 ± 147	420 ± 117
24b‘	Cg1‘	343 ± 58	164 ± 19	375 ± 152	389 ± 114
CA	CA	1417 ± 257	416 ± 142	331 ± 161	234 ± 94
DG	DG	447 ± 195	692 ± 276	348 ± 166	250 ± 103
Ce	Ce	83 ± 47	70 ± 28	284 ± 106	251 ± 112
Acb	Acb	82 ± 42	39 ± 9	370 ± 108	627 ± 295
MDT	MDT	52 ± 26	53 ± 15	330 ± 148	302 ± 84

Nomenclature of brain regions are provided in Table 1.
