# Supplementary material for: Serotonin Receptors in Areas of the Emotion Regulation Network in Human and Rat Brains—A Comparative Autoradiographic Study
Source: J Comp Neurol. 2025 Jul 16;533(7):e70068. doi: 10.1002/cne.70068 (PMC12267679; doi:10.1002/cne.70068)
Supplement: Supplementary file 2 — Supplementary Table 2: Numerical values of relative 5‐HT1A and 5‐HT2 receptor densities (in %) in areas of the emotion regulation network in the human brain and their homologs in rats. [file CNE-533-e70068-s001.pdf]

Supplementary Table 2. Numerical values of relative 5-HT_{1A} and 5-HT_2 receptor densities (in %) in areas of the emotion regulation network in the human brain and their homologs in rats.

**Species**		**5-HT_{1A} receptors**		**5-HT_2 receptors**	
Human	Rat	Human	Rat	Human	Rat
11	MO	6.38	-9.69	7.96	10.46
47	LO	21.13	-33.18	14.61	28.21
25	IL	39.93	33.91	19.18	9.94
32	Cg3	-5.82	26.51	16.15	36.62
24a	Cg2d	11.34	4.76	12.08	12.14
24b	Cg1	-21.60	-2.37	3.48	22.63
24a‘	Cg2’d	-5.69	-32.23	3.47	1.66
24b‘	Cg1‘	-21.54	-31.34	-3.70	-5.83
CA	CA	223.98	83.13	-15.16	-48.92
DG	DG	2.16	193.72	-10.68	-42.41
Ce	Ce	-81.02	-70.60	-27.05	-39.31
Acb	Acb	-81.17	-84.77	-5.10	41.90
MDT	MDT	-88.07	-77.86	-15.25	-27.08

Nomenclature of brain regions are provided in Table 1.
