# Supplementary material for: Serotonin Receptors in Areas of the Emotion Regulation Network in Human and Rat Brains—A Comparative Autoradiographic Study
Source: J Comp Neurol. 2025 Jul 16;533(7):e70068. doi: 10.1002/cne.70068 (PMC12267679; doi:10.1002/cne.70068)
Supplement: Supplementary file 3 — Supplementary Table 3: Results of the ANOVA test performed with a mixed‐effects model. [file CNE-533-e70068-s003.pdf]

Supplementary Table 3. Results of the ANOVA test performed with a mixed-effects model.

Effect	df	F-value	p-value
Main effect:			
Species	1	2.91	0.13
Receptor	1	37.55	0.00
Region	12	26.94	0.00
Interaction effect:			
Species*Receptor	1	73.79	0.00
Species*Region	12	13.78	0.00
Receptor*Region	12	37.15	0.00
Species*Receptor*Region	12	10.85	0.00
