# Supplementary material for: Serotonin Receptors in Areas of the Emotion Regulation Network in Human and Rat Brains—A Comparative Autoradiographic Study
Source: J Comp Neurol. 2025 Jul 16;533(7):e70068. doi: 10.1002/cne.70068 (PMC12267679; doi:10.1002/cne.70068)
Supplement: Supplementary file 4 — Supplementary Table 4: The results of the simple effect test with mixed‐effects model. [file CNE-533-e70068-s005.pdf]

Supplementary Table 4. The results of the simple effect test (Hawrylycz et al., 2011) of regions and the corresponding post hoc tests performed (following the significant results of the Omnibus test, Supplementary Table 3) with mixed-effects model. No post hoc tests were performed using 5-HT_2 receptor densities in the human brain because the corresponding SET was not significant.

SET of regions	Receptor	Species	*p*-value
	5-HT_{1A}	Human	0.00
		Rat	0.00
	5-HT_2	Human	0.09
		Rat	0.00
Post hoc test within neighboring regions			
5-HT_{1A}	Human	11 vs 47	0.24
		32 vs 24b	0.29
		24a vs 24b	0.02
		24a‘ vs 24b‘	0.20
		CA vs DG	0.00
	Rat	MO vs IL	0.09
		MO vs Cg3	0.20
		MO vs Cg1	0.63
		IL vs Cg3	0.63
		IL vs Cg2d	0.22
		Cg3 vs Cg2d	0.44
		Cg2d vs Cg1	0.10
		Cg2d vs Cg2’d	0.19
		Cg1 vs Cg1‘	0.22
		Cg2’d vs Cg1‘	0.97
		CA vs DG	0.00
5-HT_2	Rat	MO vs IL	0.97
		MO vs Cg3	0.11
		MO vs Cg1	0.34
		IL vs Cg3	0.10
		IL vs Cg2d	0.71
		Cg3 vs Cg2d	0.23
		Cg2d vs Cg1	0.55
		Cg2d vs Cg2’d	0.34
		Cg1 vs Cg1‘	0.06
		Cg2’d vs Cg1‘	0.76
		CA vs DG	0.80

Note: We only present partial post hoc results, which are compared between neighboring regions. It should also be noted that the homologous regions in humans and rats have different neighboring relationships. The orange color indicates that the receptor densities of CA are significantly higher than those of DG (FDR corrected), while the blue color signifies the opposite. Nomenclature of brain regions are provided in Table 1.

Hawrylycz, M., Baldock, R. A., Burger, A., Hashikawa, T., Johnson, G. A., Martone, M., Ng, L., Lau, C., Larsen, S. D., Nissanov, J., Puelles, L., Ruffins, S., Verbeek, F., Zaslavsky, I., & Boline, J. (2011). Digital atlasing and standardization in the mouse brain. *PLOS Computational Biology*, *7*(2). https://doi.org/10.1371/journal.pcbi.1001065
