# Supplementary figures and images for: Serotonin Receptors in Areas of the Emotion Regulation Network in Human and Rat Brains—A Comparative Autoradiographic Study
Source: J Comp Neurol. 2025 Jul 16;533(7):e70068. doi: 10.1002/cne.70068 (PMC12267679; doi:10.1002/cne.70068)

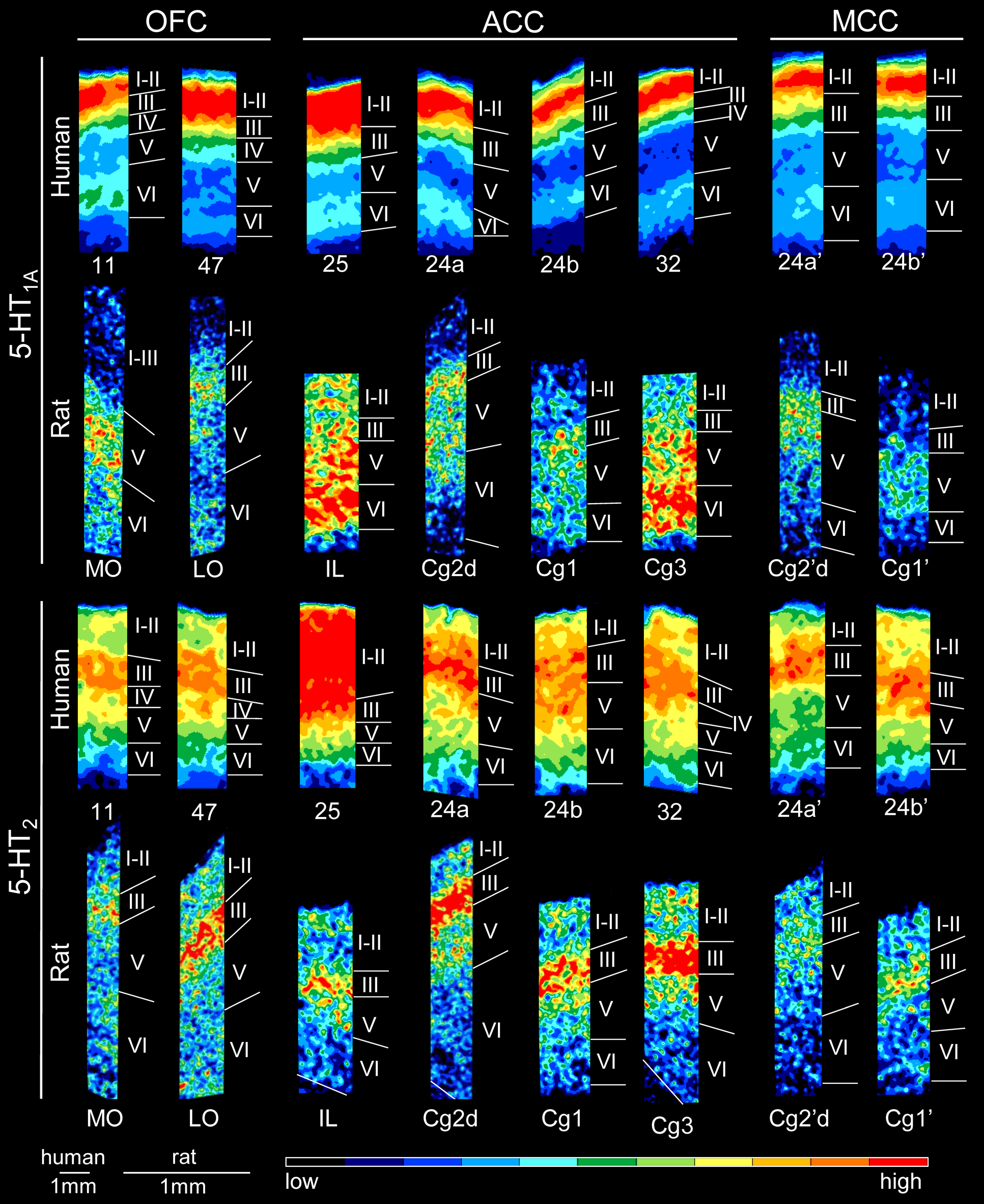

Supplement: Supplementary file 5 — Supplementary Fig. 1: Laminar 5‐HT1A and 5‐HT2 receptor distributions between the human and rat orbitofrontal and cingulate areas. [file CNE-533-e70068-s004.tif]
